# An Asian-centric human movement database capturing activities of daily living

**DOI:** 10.1038/s41597-020-00627-7

**Published:** 2020-09-08

**Authors:** Phyllis Liang, Wai Hang Kwong, Ananda Sidarta, Choon Kong Yap, Wee Kiat Tan, Lek Syn Lim, Pui Yee Chan, Christopher Wee Keong Kuah, Seng Kwee Wee, Karen Chua, Colin Quek, Wei Tech Ang

**Affiliations:** 1 Rehabilitation Research Institute of Singapore, Nanyang Technological University, Singapore, Singapore; 2 Tan Tock Seng Hospital, Singapore, Singapore; 3 School of Mechanical & Aerospace Engineering, Nanyang Technological University, Singapore, Singapore

**Keywords:** Health care, Anatomy

## Abstract

Assessment of human movement performance in activities of daily living (ADL) is a key component in clinical and rehabilitation settings. Motion capture technology is an effective method for objective assessment of human movement. Existing databases capture human movement and ADL performance primarily in the Western population, and there are no Asian databases to date. This is despite the fact that Asian anthropometrics influence movement kinematics and kinetics. This paper details the protocol in the first phase of the largest Asian normative human movement database. Data collection has commenced, and this paper reports 10 healthy participants. Twelve tasks were performed and data was collected using Qualisys motion capture system, force plates and instrumented table and chair. In phase two, human movement of individuals with stroke and knee osteoarthritis will be captured. This can have great potential for benchmarking with the normative human movement captured in phase one and predicting recovery and progression of movement for patients. With individualised progression, it will offer the development of personalised therapy protocols in rehabilitation.

## Abstract (summary)

**Table:** 

Measurement(s)	voluntary movement behavior • grip strength measurement • Pinch Strength
Technology Type(s)	motion capture system • force plates • sensor • dynamometry
Factor Type(s)	gait • Timed Get Up and Go Test • balance • reaching • key turning • hand to mouth • hand to back • hand on head • cross obstacle • folding towel • step up and down
Sample Characteristic - Organism	Homo sapiens
Sample Characteristic - Location	Asia

Machine-accessible metadata file describing the reported data: 10.6084/m9.figshare.12808187

## Background & Summary

Three-dimensional motion capture system using reflective markers provides accurate and precise data to describe joints angular movement quality^1–3^. Joint movements which are hard to be quantified using traditional methods, such as shoulder elevation^4^, scapular movements^5^, and knee valgus^6^ can be assessed with the motion capture system during functional activities. Recent studies have adopted this technique to capture the action during activities of daily living (ADL) and functional tasks in both upper limbs^4,7,8^ and lower limbs^9^. Clinically, it is important to assess the level of impairment and function in people with movement impairment, such as individuals with stroke, to track the level of recovery and determine the efficacy of treatments. Most of the standardised clinical assessments summarise the patients’ performance with a total score or collapse a time-varying movement data into a point estimate. In doing so, most of the information including variability in temporal and spatial domains cannot be captured. As a result, these tools are insensitive in picking up subtle changes in motor performance and detecting abnormalities in patients with high function^10^.

One of the objectives of movement analysis is to determine whether a movement deviates from the average^11,12^. Knowledge about normal kinematics in extremities and trunk during functional tasks could provide a basis to evaluate the level of recovery and effect of rehabilitation interventions, as the regain of normal movement pattern is always treated as a benchmark of motor recovery^7,13^. For example, Aboelnasr *et al*.^14^ assessed the quality of reaching movement in children with cerebral palsy by comparing their movement with normally developing children. In addition to the overall completion time, the level of jerkiness and spatial inaccuracy of hand control were able to be captured and compared between the two groups^14^. Thus, this approach could maximise the information gained from clinical assessments^15,16^.

There are a few open access electronic databases that provide kinematic and/or kinetic data on human participants, including HuMoD Database^17^, Berkeley Multimodal Human Action Database^18^, CMU Graphics Lab Motion Capture Database^19^ and KIT Whole-Body Human Motion Database^20^. The HuMoD Database^17^ provides both raw and processed kinematic and kinetic data as well as electromyographical measurements. This database contains motion capture data of 8 lower limbs tasks (e.g. walking, kicking ball, squatting) which were performed by one female (27 yrs, 161 cm, 57 kg) and one male participant (32 yrs, 179 cm, 85 kg). The Berkeley Multimodal Human Action Database^18^ contains 11 actions performed by 12 participants (7 male and 5 female). 11 of these participants were between 23–30 years old, with one elderly participant. This database mainly comprises of actions with high dynamics such as jumping jacks, throwing and hand clapping. The CMU Graphics Lab Motion Capture Database^19^ provides a wide range of motion capture data, including the interaction between human participants, sports activities (e.g. basketball, dance) and ADLs (e.g. sweeping floor, washing window). There were a total of 144 participants and each participant generated different motions. The ongoing KIT Whole-Body Human Motion Database^20^ captures the motions from 224 participants (with 106 males, 37 females and the rest of the participants without gender specified) and 127 different objects (e.g. cup, basket) and environmental elements (e.g. staircase, seesaw) with which the participant is interacting. These open access databases have recorded numerous motion capture data of healthy individuals. Nevertheless, none of these databases attempted to capture the motion of the Asian population. Ethnicity has been reported to be influential for both body proportion and body composition^21,22^. In addition, anthropometry differences exist between ethnic groups^23,24^. Within Southeast Asia, populations from neighbouring countries have different body dimensions^23,25^, and so do people of historically highly associated ethnic groups in East Asia^24^. Up to half of the body dimensions measured were significantly different between the Singaporean and Indonesian adult^25^, and the former had the greatest stature compared to other Asian populations^26^. Differences were also found when comparing the Singaporean Chinese elderly population to those in Malaysia and Chinese living in Beijing^26^. Thus, establishing a movement database for Asian population is warranted.

The current work aims to establish a normative movement database containing kinetic and kinematic data of 500 healthy adults. It represents our latest effort in building an Asian-centric movement database that focuses on the activities of daily living. Kinematics and kinetics data of 12 upper and lower body tasks were captured. These tasks were either selected from a standardised assessment tool or were representative of daily functional activities such as reaching to grasp an object, turning a key in a lock and walking. The large sample size allows us to capture variations of normal movement patterns, which could provide sufficient data for data-driven healthcare and rehabilitation services, and building machine learning models.

The study protocol is described in this article. Data from 10 participants which captured using the described protocol are available^27^.

## Methods

### Participants

A total of 500 healthy participants (aged 21-80) of Asian ethnicity will be recruited for this study. Exclusion criteria include: (1) declared prior neurological conditions, surgeries, or medical conditions that need active medical or therapy intervention in the last three months, (2) declared depression or mental health issues affecting daily task performance, (3) declared visual problems that resulted in a recent accident, fall, or near-fall, (4) having skin lesions or known skin allergies that would hinder markers placement, (5) inability to participate normally in daily living tasks due to pain, or (6) pregnancy. In this paper, we report the protocol details and the data from ten healthy participants who have completed the trial. See Table 1 for basic demographics and body measurements. This study was approved by the Nanyang Technological University Institutional Review Board (IRB-2018-04-014). Recruitment methods include flyer advertisements posted in public areas, word of mouth, visiting organisations to share information about the study, exhibition booths at events such as conferences and various community centres. There will be an even distribution of participants for each age group range and gender. The proportion of ethnic group will mirror the ethnic group distribution in Singapore (Chinese: 75%, Malay: 13%, Indian: 9%, others: 3%)^28^. To ensure that all ethnic groups are captured, recruitment will include sharing information about the study at specific ethnic group associations.

**Table 1:** Basic demographics and body measurements of participants.

Participant	1	2	3	4	5	6	7	8	9	10
Age range	41–50	41–50	61–70	21–30	31–40	31–40	51–60	51–60	51–60	71–80
Gender	Male	Female	Female	Male	Male	Male	Male	Female	Female	Female
Hand dominance	Right	Right	Right	Right	Right	Left	Right	Right	Right	Right
Mass (kg)	69.9	52.9	59.0	75.1	60.2	65.4	77.3	42.3	53.0	51.3
Stature (mm)	181	164	161	182	166	169	178	154	159	151
Ethnic group	Chinese	Chinese	Chinese	Chinese	Chinese	Chinese	Indian	Chinese	Chinese	Chinese

### Experimental set-up and equipment

All trials were conducted in the motion capture laboratory of Rehabilitation Research Institute of Singapore (RRIS).

#### Motion capture system

**Fig. 1:**
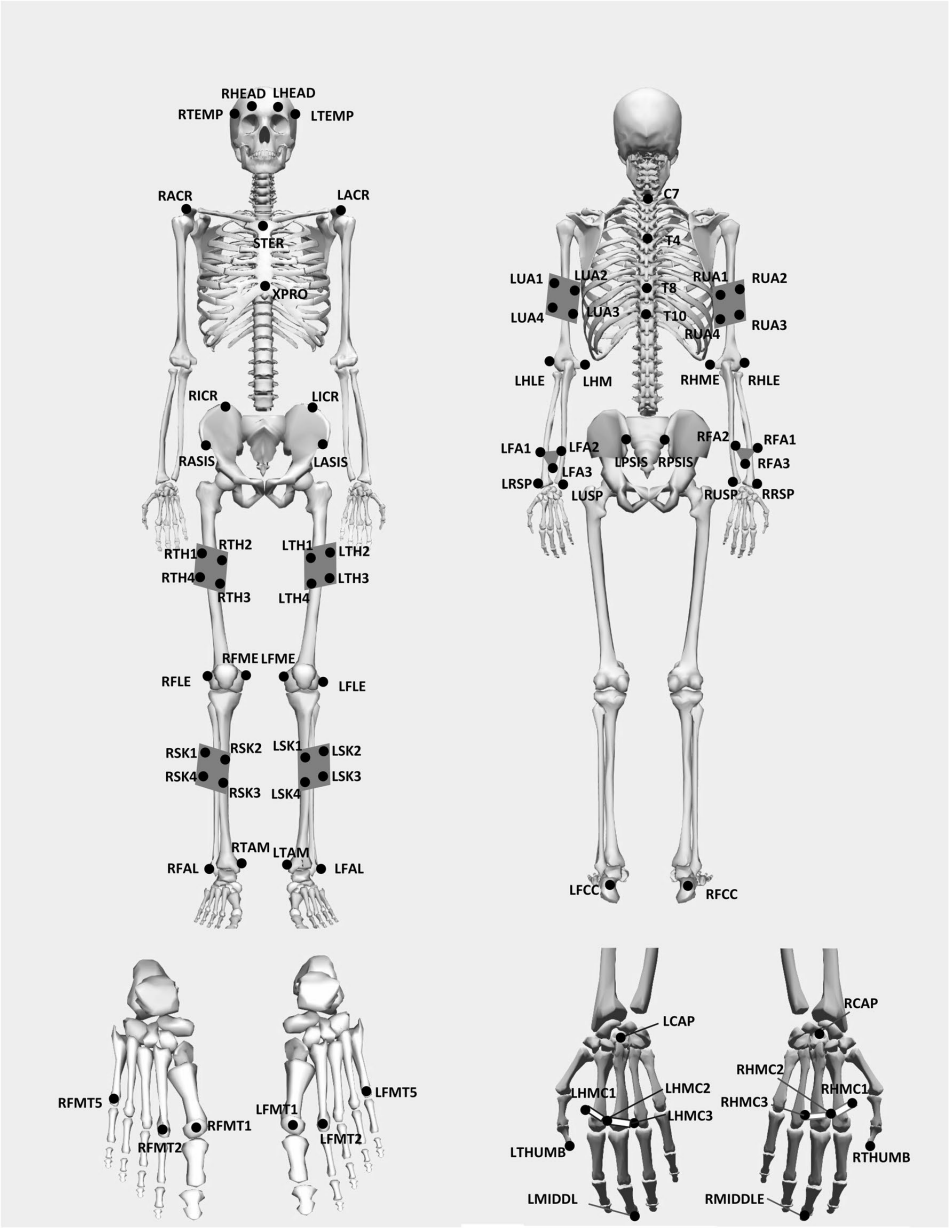
Marker placement set (skeleton from OpenSim).

Three-dimensional human movement data was captured using sixteen 2 megapixels Miqus M3 motion capture system (Qualisys, AB, Sweden), with a field of view (FOV) of 64 × 41 degrees. Retro reflective optical markers were placed on the body of a participant according to the marker placement set (Fig. 1). The marker set was based on a modified Calibrated Anatomical System Technique (CAST), which has a diameter of 12.5 mm (for the body) and 10 mm (for finger tips). The motion capture cameras emit infrared strobe, which will be reflected back by the markers. In this way, the cameras are able to capture and record the movement trajectory of the body. The Qualisys Track Manager (QTM) v2019 served as an integrated software interface for seamless and easy-to-use data recording. QTM is capable of synchronizing the cameras with external devices, such as force plates and other electromechanical sensors. The trajectories of the markers and data from external devices were captured synchronously at 200 and 2000 Hz rate respectively.

#### Force plates

**Fig. 2:**
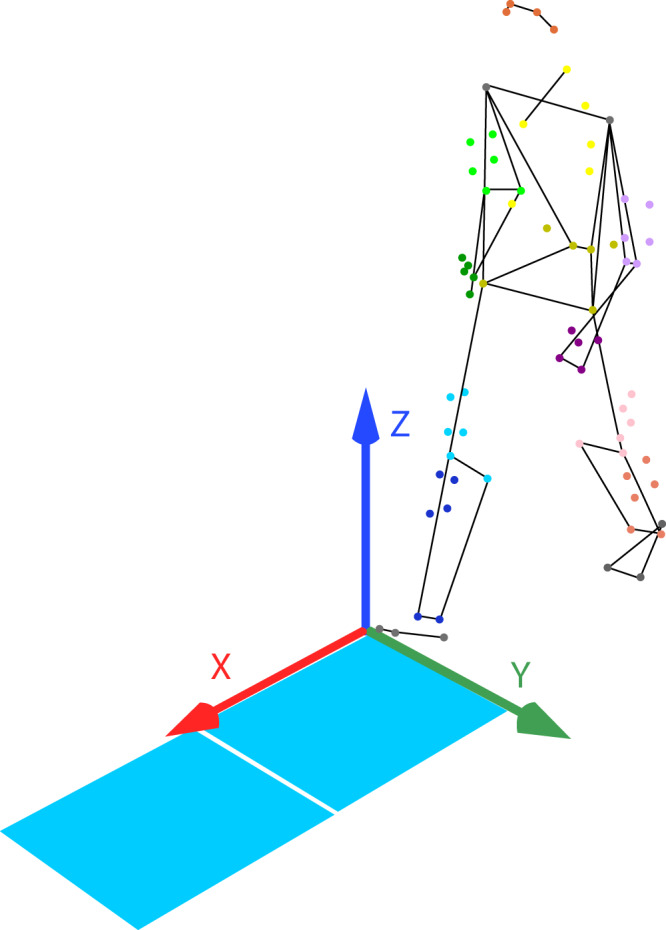
Diagram representing the force plates with the directions of the axes.

Two 60 × 50 x 5 cm force plates (Type 9260AA6, Kistler, Switzerland) were positioned on the floor to record the three-dimensional ground reaction force during lower limb tasks. (See Fig. 2).

#### Other equipment and sensorised items

**Fig. 3:**
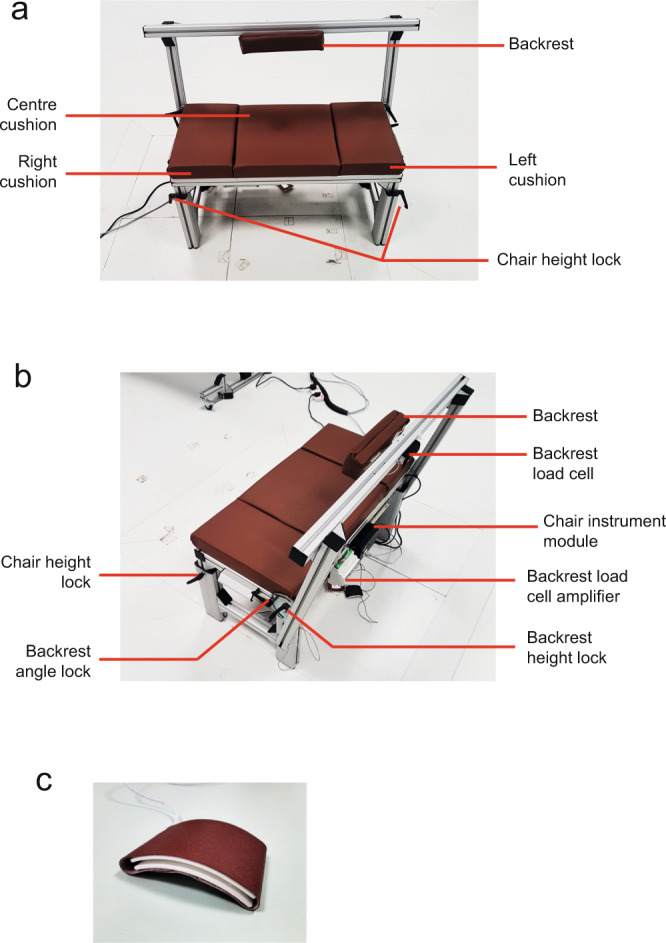
(**a**) Front view of sensorised chair. (**b**) Angled view of sensorised chair. (**c**) Lap sensor.

Participant was seated in front of a table on a chair that was designed in-house. The sitting area of the chair was about 39 × 83 cm. The chair height could be adjusted between 41 – 59 cm, and its backrest could also be adjusted to different angles and heights to suit different participant’s torso height. The sensorised chair had a tension and compression load cell (FSH04207, FUTEK Inc., USA) installed at the backrest of the chair. See Fig. 3a,b. The chair was also connected with two mini push-button switches that were embedded in a cover to be used as left and right lap sensors (Fig. 3c).

**Fig. 4:**
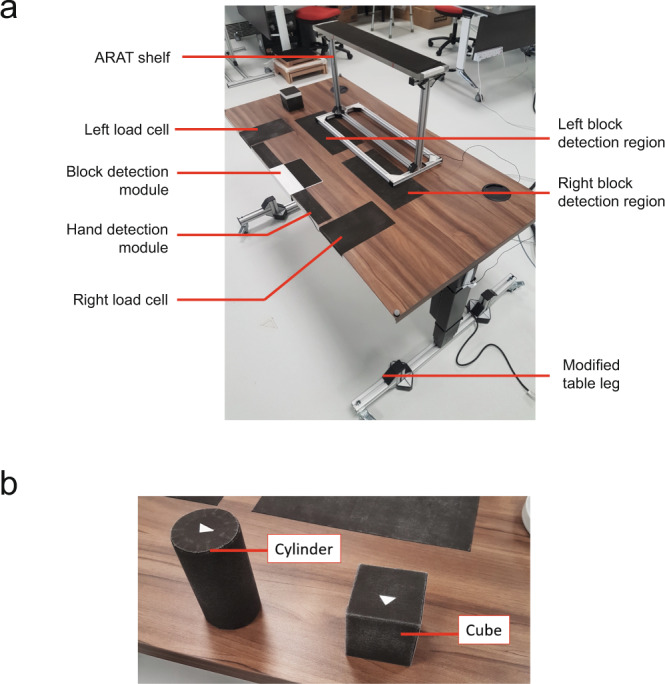
(**a**). Sensorised table with cube detection plate and ARAT shelf. (**b**) Cylinder and cube used for the tasks.

The AmpDesk table (ErgoEdge, Singapore) with an adjustable height was customised for the study. Two load cells (C2G1, Pavone Sistemi, Italy) were embedded to measure force exerted on the table by each hand during upper limb trials. Conductive fabric (EeonTex, SparkFun Electronics) was affixed over different section of the table, a cube detection plate and also the Action Research Arm Test (ARAT) shelf (Fig. 4a). These conductive fabrics were connected to programmed microcontrollers and acted as contact capacitive touch sensors that allowed us to measure the precise moment an object comes into or is removed from contact with the hand. Conductive fabric also covered the contact surface of a cube and cylinder (Fig. 4b) used in the protocol during Forward-Reach Grasp and Lateral-Reach Grasp tasks respectively. This enabled the capacitive touch capability of the fabric on the table to be extended to the cube and cylinder.

**Fig. 5:**
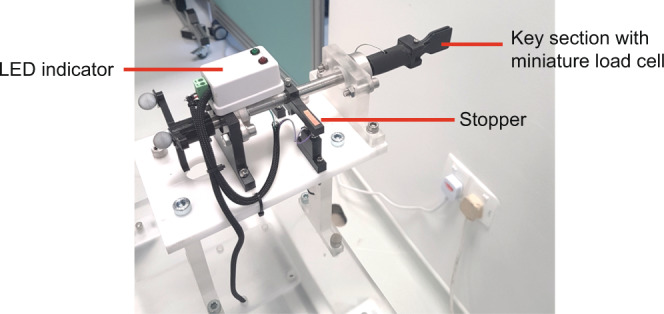
Key rig.

A fixture with a key-like object was designed to capture supination and pronation of the forearm (Fig. 5) during a task that required the participant to simulate locking and unlocking a door with key. The key-like object had a sub miniature load button (FSH03879, FUTEK Inc.) embedded inside to measure the pinch force during the task. The height of the key was 97 cm from the ground which was within the Building and Construction Authority of Singapore’s recommended height of 90 cm to 110 cm^29^.

#### Dynamometer

Jamar Plus + Digital Hand Dynamometer and Jamar Digital Pinch Gauge (Sam-mons Preston, Bolingbrook, IL, USA) were used to assess grip strength and lateral pinch strength.

### Procedure

Participants were encouraged to wear appropriate attire for the data collection (e.g. shorts or exercise tights and a tight fitting singlet). Clothing should be dark in colour with minimal reflective material. In cases where participants did not have appropriate attire, the research team provided clothing.

**Fig. 6:**
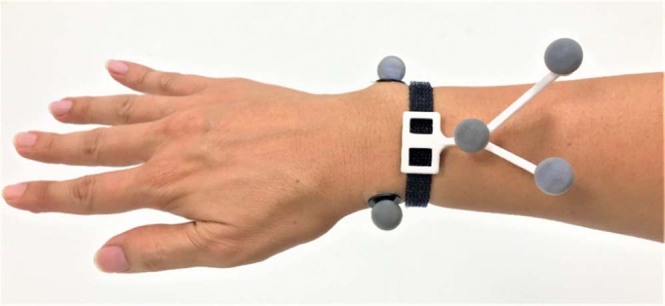
Rigid body to capture forearm movements.

Basic anthropometry, demographic and lifestyle information were taken, e.g. height, weight, marital status, level of education. Basic strength tests for the upper limb was conducted using the Jamar dynamometer. Markers were placed onto the participant’s body according to the Marker placement protocol illustrated in Fig. 1. For the forearm markers, they were affixed onto a rigid body that was modified to capture forearm movements more effectively (See Fig. 6)^30^.

After the markers were placed on the participant, a static trial was taken so that all markers could be seen. A static calibration pose (shoulder abduction 45 degrees, elbow fully extended, palms facing forward) was held for at least two seconds.

Following the static trial, participants went through a series of 12 tasks, consisting of 6 upper limb and 6 lower limb tasks. The sequence of the task was based on block randomization, which was determined by throwing a dice prior to the trial. This randomization was done to minimize bias due to mental and physical fatigue during the trial. Table 2 details the sequence of tasks in each block.

**Table 2:** Tasks in each randomization block.

Block 1	Block 2	Block 3	Block 4	Block 5	Block 6
1. Folding towel	1. Forward reach-grasp	1. Hand to back	1. Balance test	1. Step up and down	1. Timed Up and Go
2. Forward reach-grasp	2. Lateral reach-grasp	2. Hand to mouth	2. Step up and down	2. Cross obstacle	2. Key turning
3. Lateral reach-grasp	3. Hand to mouth	3. Hand on head	3. Cross obstacle	3. 10 m walk	3. Step up and down
4. Hand to mouth	4. Hand on head	4. Forward reach-grasp	4. 10 m walk	4. Balance test	4. Cross obstacle
5. Hand on head	5. Hand to back	5. Lateral reach-grasp	5. Key turning	5. Key turning	5. Balance test
6. Hand to back	6. Folding towel	6. Folding towel	6. Timed Up and Go	6. Timed Up and Go	6. 10 m walk
7. Timed Up and Go	7. Timed Up and Go	7. Timed Up and Go	7. Hand to back	7. Folding towel	7. Hand to back
8. Key turning	8. Key turning	8. Key turning	8. Hand to mouth	8. Forward reach-grasp	8. Hand to mouth
9. Balance test	9. Step up and down	9. Cross obstacle	9. Hand on head	9. Lateral reach-grasp	9. Hand on head
10. Cross obstacle	10. Balance test	10. Step up and down	10. Forward reach-grasp	10. Hand to mouth	10. Forward reach-grasp
11. Step up and down	11. Cross obstacle	11. Balance test	11 .Lateral reach-grasp	11. Hand on head	11. Lateral reach-grasp
12. 10 m walk	12. 10 m walk	10 m walk	12. Folding towel	12. Hand to back	12. Folding towel

In each task, the participant was first provided with a short explanation and shown task demonstrations by an experimenter. This was followed by a few familiarization and practice trials. The actual trial would commence once the participant confirmed that the instructions given had been understood.

For the upper limb tasks, participants repeated each action for 6 times per limb, that is, the dominant then non-dominant limb. For the lower limb tasks, participants repeated each action for 3 times per limb. A 10-15 minute break was given to the participants between upper and lower limb tasks. The 12 tasks were selected based on expert advice from occupational therapists and physiotherapists. Each task represented an important movement required to complete everyday functional tasks.

For all the six upper limb tasks detailed below, the chair was set up so that the participant’s hip and knee flexion was at approximately 90 degrees and participants were instructed to keep their feet flat on the ground throughout the tasks^31^. For tasks three to five, participants were instructed to keep their back against the backrest.

**Fig. 7:**
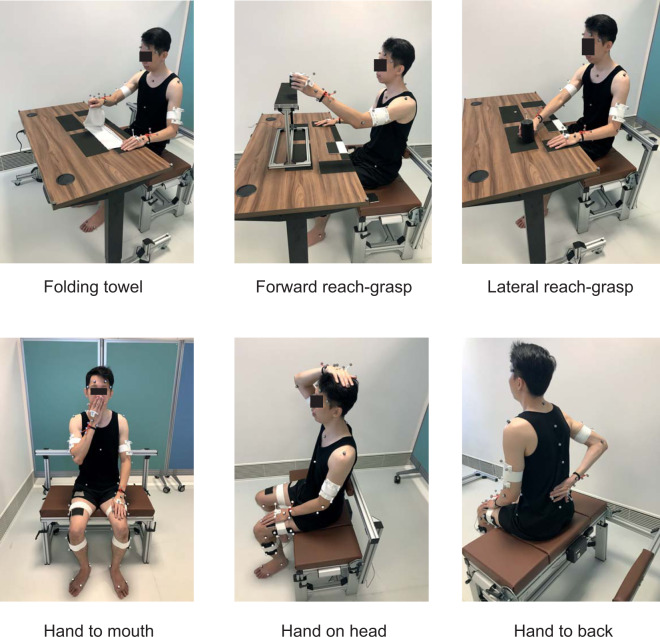
Upper limb tasks.

**Fig. 8:**
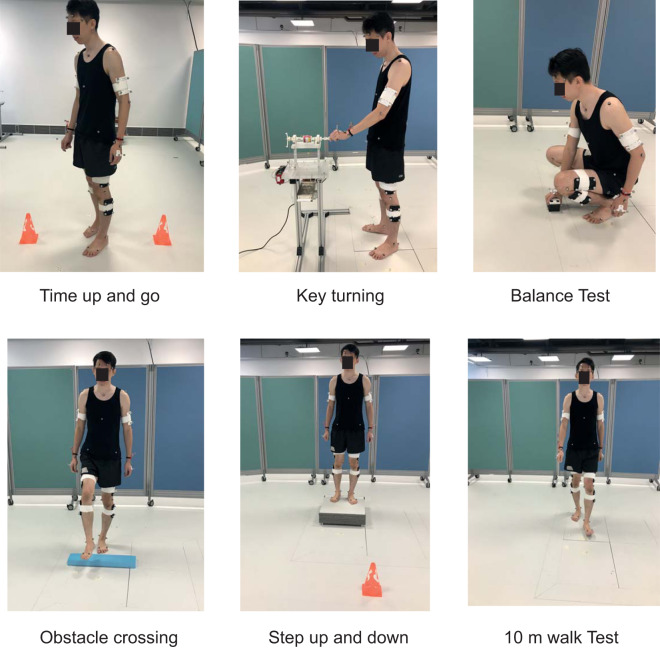
Lower limb tasks.

The following section describes the 12 tasks in the protocol. Figures 7 and 8 are images of the task being performed by an internal staff of the research institute. Written consent was obtained from the staff for the use of his image.

1. Folding Towel
  Folding towel was a test extracted from a standardised assessment known as the Wolf-Motor Function Test^32^. This task assessed the participant’s ability in manipulating objects used with daily living. Bilateral limb coordination could be evaluated since synchronization of hands movement was required in performing this task. The participants were required to fold the towel, that has a size of 55.5 x 29 cm, once from top to bottom, and then fold it another time from one side to another.

2. Forward Reach-grasp
  This task was extracted from Action Research Arm Test^31^. This test assessed the participant’s ability in manipulating the cube including controlled grasp and release. The voluntary control of shoulder flexion combined with elbow extension was also assessed. In patient with brain lesions, this movement pattern is sometimes impaired due to the abnormal shoulder-elbow coupling. The participants were required to grasp a cube (7.5 cm^3^) with conductive fabric on the table and transport it to the top of the shelf. The cube would then remain on the shelf while the hand would return to the starting position. The grip used to grasp the cube had to be with the forearm in a neutral position and a grip over the top of the block was not permitted. The experimenter brought the block down to starting position and participant waited for the cue from the experimenter to repeat the subsequent movement.

3. Lateral reach-grasp
  This test assessed the participant’s ability in performing a cylinder grip and transferring an object across the body midline. Truncal compensation would also be observed since it is one of the indicators of dynamic sitting balance^33^. The starting position was similar to the forward reach-grasp task. Here, the participant would reach across the midline to grasp the object positioned in line with the opposite shoulder at maximum reach. Once grasped, the object was transported horizontally to be in line with the shoulder of the arm being tested. The positioning of the object was marked prior to the task to ensure it was consistent across the trials. During the task, the position of the supporting hand remained on the table.

4. Hand to mouth
  This task was extracted from Action Research Arm Test^31^ which simulated the gross movement during feeding. The activity was to lift the hand, place the hand on the mouth and then back to the initial position on the lap.

5. Hand to head
  This task was extracted from Action Research Arm Test^31^ which simulated the gross movement during hair combing. Participants lifted up the hand to place it on the top of the head, and brought it back to the initial position on the lap.

6. Hand to back
  This task simulated the movement during back washing. This movement is important, for example, for showering or personal hygiene. The task began with the hands pronated on the lap. The participants moved their hand being tested towards their lower back, targeting the middle of their lower back with their palm. During the task, the position of the supporting hand remained on the lap.

7. Timed Up and Go (TUG)
  The TUG is one of the standardised tests in assessing functional mobility in clinical setting^34–36^. Multiple transitional movements, including sit-to-stand and turning, were involved in the test which make the performance of the test highly relevant to the locomotion function in ADLs^36^. The participant was seated and the starting position of the feet was on the force plate.

8. Key turning
  Participants performed this task in standing, with the body erect and feet at hip distance apart, facing towards the key rig and both arms relaxed in neutral position at side of body. The participants brought the hand to hold the key, and turned the key in a clockwise direction to simulate locking the door followed by an anticlockwise direction to simulate unlocking the door.

9. Balance
  This task was extracted from the Berg Balance Scale^37^. The task challenged the participant’s anticipatory postural adjustment and the ability to move the center of gravity in the superoinferior direction. A previous study has shown that this item demonstrated the strongest power in differentiating faller from non-faller in elderly^38^. This item is rated on a 5‐point ordinal scale in the Berg Balance Scale clinically. In this particular task, participants to stand upright with hands relaxed at their sides and each foot on either side of the force plate. Following a cue, they had to bend downwards to pick up an object off the floor. Once the object was grasped, they had to return to the standing position and pass the object to the experimenter. Both feet remained in the same position throughout unless the participant self-initiated a compensatory strategy by adjusting their foot position in order to perform the task.

10. Obstacle crossing
  This task was used to simulate crossing an obstacle, for example, in a bathroom. The ability to clear foot from floor and the coordination between stance and swing limb would be assessed in this task^39^. The participant began standing facing the direction of the walkway with both hands relaxed at the side. Participant would step over the obstacle placed across the walkway with one foot at a time. The movement was completed when both feet were on the other side of the obstacle.

11. Step up and down
  Stepping up and down is essential for community ambulation and functional independence. The task can be demanding for people with motor impairment since it required high level of dynamic balance ability and at the same time challenging their concentric and eccentric lower-limbs muscle strength^40^. The task involved a standing platform (60 cm length x 50 cm width x 15 cm height) that was placed on the force plate area. During the task, the participants were required to step from the flat ground onto the platform one leg at a time. They would then walk to the edge of the platform before stepping off the step. This task simulated daily activities involving crossing a kerb.

12. 10 m Walk
  The 10 m Walk test is considered one of the core assessments for lower-limb function in rehabilitation^41,42^. Kinetic and kinematics information obtained from this walking task would allow the participant’s gait to be analysed. Clinically, gait analysis has been used to diagnose pathology and evaluate the effectiveness of intervention^43^. The participant was required to walk a total distance of 10 metres, which was mapped out with a demarcated start and finish line along the walkway. Throughout the task, the participant was instructed not to look at the ground, where the force plates were installed, but to look straight ahead to a far distance object.

## Data Records

Raw data captured consisted of the marker coordinates that were synchronized with information recorded by the force plate and sensorised items. Markers were labelled and missing data were gap filled using the QTM software (Qualisys Track Manager, proprietary software that comes with Qualisys MOCAP). Data were saved in.qtm format (which is a proprietary file format for Qualisys) but converted to c3d format for data sharing. c3d format is widely supported in many motion capture systems (Qualisys, Vicon), analysis software (Visual3D), or commonly used language library (python, R, Matlab). The benefit of saving in c3d format is that most information can be saved in a single file (marker trajectories, analog data, force data). 10 trials data are publicly available at NTU Dataverse (10.21979/N9/7VF22X)^27^.

One unique folder was created for each participant which contain data files (in c3d format) from both lower limb and upper limb tasks and the static pose. The folders were labelled ‘SNxxx’, where xxx is an integer value representing the participant number (for example SN001). Each file was systematically named as ‘SNxxx_<file-number>_<abbreviated-task-name>_<side><recording-number>’. For example, SN001_0028_towel_R02 denotes the data file for participant SN001 performing the folding towel task using the right arm in the second recording. Full lists of task name used in file naming are shown in Table 3.

**Table 3:** Tasks naming of file.

Task naming of file	Actual task
static	static posture
towel	folding towel
grasp	forward reach-grasp
lateral	lateral reach-grasp
mouth	hand to mouth
head	hand on head
back	hand to back
tug	timed up and go
key_stand	key turning
balance	balance test
kerb	cross obstacle
step_down	stepping down
step_up	stepping down
10 m	10 m walk

Each c3d file contained multiple data stored into a single file: marker trajectories data (under Video Data group), sensor data (under Analog Data group, see Table 4 for full list of sensors used) and force data (under Analog Data group, see Table 5 for full list of force signal). Important parameters were saved under respective group as well (POINT, ANALOG, FORCE_PLATFORM). For example POINT:RATE referred to the sampling rate used in MOCAP. Force data consisted of raw output signal from the sensor embedded in the force plate. Ground reaction force and center of pressure (COP) were derived from output signal using the formula provided (https://isbweb.org/software/movanal/vaughan/kistler.pdf).

**Table 4:** Sensor naming in c3d file (under Analog data).

Sensor naming in c3d file	Actual sensor
1_Block	cube detection plate (Fig. 3a)
2_Hand	hand detection region (Fig. 3a)
3_Table_left	left block detection region (Fig. 3a)
4_Table_right	right block detection region (Fig. 3a)
5_Shelf	ARAT shelf (Fig. 3a)
6_Loadcell_left	left load cell (Fig. 3a)
7_Loadcell_right	right load cell (Fig. 3a)
8_Lap_left	left lap (Fig. 2c)
9_Lap_right	right lap (Fig. 2c)
10_Chair	backrest load cell (Fig. 2b)
13_Key_Pinch	key section with miniature load cell (Fig. 4)

**Table 5:** Force component naming in c3d file (under Analog data) (refer to https://isbweb.org/software/movanal/vaughan/kistler.pdf for naming convention and formula used).

Force component naming in c3d file	Force signal
Channel_01	force plate 1: fx12
Channel_02	force plate 1: fx34
Channel_03	force plate 1: fy14
Channel_04	force plate 1: fy23
Channel_05	force plate 1: fz1
Channel_06	force plate 1: fz2
Channel_07	force plate 1: fz3
Channel_08	force plate 1: fz4
Channel_09	force plate 2: fx12
Channel_10	force plate 2: fx34
Channel_11	force plate 2: fy14
Channel_12	force plate 2: fy23
Channel_13	force plate 2: fz1
Channel_14	force plate 2: fz2
Channel_15	force plate 2: fz3
Channel_16	force plate 2: fz4

## Technical Validation

To ensure that the data collection procedure is reliable, the markers were placed by researchers who are trained by clinicians. The clinicians provided training and hands-on guidance and ensured that the researcher is competent to do so on their own.

The reliability of marker placement had been evaluated in a separate trial to ensure the data collection procedure is repeatable. Two testers placed the anatomical markers on the same participant in an alternative order twice. Thereby, the inter-tester reliability and the intra-tester reliability of the two testers were assessed. The absolute mean differences in joint angle and the R^2^ deduced from the Linear Fit Method were used to evaluate the reliabilities. The inter-rater reliability was assessed by comparing the gait kinematics after the first trial of markers placement of each tester.

The participant performed the 10 m walking test and 10 strides from 3 – 4 records were extracted for the analysis of the reliability of gait kinematics. The 10 m walking test was selected because the reliability of the 3-dimensional gait kinematics has been well studied. For example, the and minimal detectable changes^44^ and similarity index^45^ of gait kinematics have been suggested previously. Therefore, the results of our technical validation study could be compared with the results of the existing literature.

The absolute mean differences in lower-limbs joint angles at heel stride, toe-off, maximal flexion and maximal extension were used to assess the repeatability of the data collection procedure.

The minimal detectable changes of gait kinematics reported by Meldrum *et.al*.^44^ was used to define the limit of agreement. All absolute mean joint angles differences were smaller than the reported minimal detectable changes (Tables 6–8)^44^.

**Table 6:** Intra-tester differences in lower-limb joint angles on sagittal plane for tester 1.

	Heel strike		Toe off		Max. flexion/dorsiflexion		Max. extension/plantarflexion		Minimal detectable changes reported by Meldrum *et al*.	
	mean differences/degree	SD of differences	mean differences/degree	SD of differences	mean differences/degree	SD of differences	mean differences/degree	SD of differences	Max. flexion/dorsiflexion	Max. extension/plantarflexion
Right ankle	0.4	0.4	3.0	0.8	1.3	0.8	3.1	0.9	8.1	10.6
Right knee	0.4	0.5	0.1	0.0	1.6	1.0	0.6	1.8	6.3	6.0
Right hip	0.6	0.4	0.1	0.1	0.0	0.0	1.0	0.7	8.3	7.6
Left ankle	0.2	0.3	2.3	0.9	0.3	0.2	2.2	0.9	8.1	10.6
Left knee	1.4	0.9	2.7	1.3	2.9	2.3	0.8	2.8	6.3	6.0
Left hip	1.6	1.1	1.5	1.4	0.7	0.8	2.4	2.2	8.3	7.6

**Table 7:** Intra-tester differences in lower-limb joint angles on sagittal plane for tester 2.

	Heel strike		Toe off		Max. flexion/dorsiflexion		Max. extension/plantarflexion		Minimal detectable changes reported by Meldrum *et al*.	
	mean differences/degree	SD of differences	mean differences/degree	SD of differences	mean differences/degree	SD of differences	mean differences/degree	SD of differences	Max. flexion/dorsiflexion	Max. extension/plantarflexion
Right ankle	1.3	1.0	7.2	3.5	0.7	1.0	7.0	3.4	8.1	10.6
Right knee	1.1	1.2	3.2	2.5	1.4	1.5	0.5	1.3	6.3	6.0
Right hip	0.7	0.5	0.8	0.8	1.1	1.8	1.0	1.0	8.3	7.6
Left ankle	2.4	3.1	0.3	0.2	2.7	2.0	0.3	0.1	8.1	10.6
Left knee	1.6	1.6	2.9	2.1	4.3	4.4	0.1	0.4	6.3	6.0
Left hip	1.4	0.9	0.3	0.2	1.1	1.3	1.7	1.6	8.3	7.6

**Table 8:** Inter-tester differences in lower-limb joint angles on sagittal plane.

	Heel strike		Toe off		Max. flexion/dorsiflexion		Max. extension/plantarflexion		Minimal detectable changes reported by Meldrum *et al*.	
	mean differences/degree	SD of differences	mean differences/degree	SD of differences	Max. flexion/dorsiflexion	Max. flexion/dorsiflexion	mean differences/degree	SD of differences	Max. flexion/dorsiflexion	Max. extension/plantarflexion
Right ankle	0.3	0.2	6.5	2.7	8.1	8.1	6.2	2.5	6.3	6.0
Right knee	1.7	1.6	3.5	2.5	6.3	6.3	0.9	2.8	8.3	7.6
Right hip	2.2	1.4	3.9	2.5	8.3	8.3	2.3	1.5	8.1	10.6
Left ankle	1.4	1.6	0.4	0.2	8.1	8.1	0.4	0.2	6.3	6.0
Left knee	1.1	0.8	1.3	0.7	6.3	6.3	0.1	0.4	8.3	7.6
Left hip	3.0	2.0	1.1	0.8	8.3	8.3	3.4	3.7	8.1	10.6

The Linear Fit Method proposed by Iosa *et.al*.^45^ was used to assess the waveform similarity in gait kinematics. The coefficient of determination R^2^ that deduced from the Linear Fit Method indicated the goodness of fit. Since there is no consensus on defining the acceptable value of R^2^, values of the lower boundary of 95% CI of the R^2^ reported by Iosa *et.al*.^45^ were used to guide the interpretation of the R^2^ in this technical validation study. The R^2^ for sagittal hip, knee, and ankle kinematics were close to 1 (Table 9) in both inter-rater and intra-rater conditions, and indicated an almost perfect waveform similarity. Besides, all R^2^ values were greater or equal to the lower boundary of 95% CI of the R^2^ reported by Iosa *et.al*.^45^ (Ankle: 0.86, Knee: 0.96; Hip:0.98).

**Table 9:** The coefficient of determination R^2^ in lower-limb joint angles on sagittal plane for intra-rater and inter-rater conditions.

Conditions	The coefficient of determination R^2^ deduced from the Linear Fit Method					
	Right ankle	Right knee	Right hip	Left ankle	Left knee	Left hip
Intra-rater (tester1)	0.986	0.998	0.998	0.994	0.999	0.999
Intra-rater (tester2)	0.990	0.999	0.980	0.999	0.998	0.994
Inter-rater	0.979	0.996	0.995	0.984	0.998	0.994
